# A Regional Health Care System Partnership With Local Communities to Impact Chronic Disease

**Published:** 2004-09-15

**Authors:** Marcus Plescia, Dennis R Joyner, Teresa L Scheid

**Affiliations:** Carolinas Community Health Institute, Carolinas HealthCare System and North Carolina Division of Public Health; Carolinas Community Health Institute, Carolinas HealthCare System; Department of Sociology, University of North Carolina at Charlotte, Charlotte, NC

## Abstract

Regional health care systems have significant opportunities to adopt community-oriented approaches that impact the incidence and burden of chronic disease. In 1998, a vertically integrated, regional health care system established a community health institute to identify, understand, and respond to health needs from a community perspective. The project was implemented in four communities (two rural counties, a rural/urban transitional county, and an inner-city community) using five steps: 1) support or form a local community coalition; 2) hire and support a local coordinator; 3) prepare a formal community assessment; 4) fund locally designed interventions; and 5) evaluate each project.

In four narrative case studies, we present the steps, challenges, and common principles faced at the local level by Carolinas Community Health Institute. The case studies were prepared using three data sources: reviews of written documents, interviews with the seven-member steering committee, and interviews with six key informants from each county. Data were coded and analyzed using standard qualitative software to identify common themes and sources of variance between cases.

The project model was generally well accepted. Local autonomy and domain disputes were challenges in all four sites. Funding for local projects was the most frequently cited benefit. The project was successful in increasing local capacity and supporting well-designed interventions to prevent chronic disease. This approach can be used by large health care systems and by other organizations to better support local health initiatives.

## Background

The tremendous growth and consolidation of regional health care systems across the United States have challenged hospital systems to understand the multiple resources and needs of the diverse communities they serve. Large vertically integrated systems now have opportunities to reach beyond the traditional medical model and adopt community-oriented approaches that impact the incidence and burden of chronic disease (1-6). While there has been considerable study of coalitions and networks that have been formed to address community health issues collaboratively (7-14), more information is needed on the role and experience of regional health care systems in developing partnerships and supporting local health promotion in the communities they serve.

The purpose of this case study evaluation is to describe the experience of a large regional health care system in implementing a community health promotion planning model in four diverse community settings. In this article, we present a framework based on our findings to guide collaborative efforts between health care systems and local communities to prevent and reduce the impact of chronic disease.

## Context

In 1998, Carolinas HealthCare System (CHS), a vertically integrated, nonprofit health authority, established Carolinas Community Health Institute (CCHI) to assist communities in developing effective health promotion and disease prevention initiatives. Funding for CCHI was provided by a federal grant from the Health Resources and Services Administration's (HRSA's) Office of Rural Health Policy, and the project was overseen by a seven-member steering committee. The structure of the CCHI project, based on established community health planning theory, involves five steps: 1) support or form a local community coalition; 2) hire and support a local coordinator; 3) prepare a formal community assessment; 4) fund locally designed interventions; and 5) evaluate each project. Over the six-year course of the project, approximately half of the grant funds were distributed to the counties in the form of salaries for local coordinators and funding for specific, locally designed health promotion projects. CCHI recently expanded to include a fifth site, and funding is now being negotiated with a local foundation.

The initiative was implemented in four geographical areas within the region: a small rural county; a midsize rural county; a transitional rural-urban county; and a high-density urban community in a metropolitan county. Demographic data and burden of chronic disease indicators are summarized for each area in Table 1.

## Methods

We used three data sources to document and describe each case study (19). All data were collected by a research assistant who had not been previously involved with the CCHI project. Narrative case reports were prepared for each of the four projects using all three data sources. The case study materials were analyzed collectively to draw cross-case conclusions about community capacity and readiness to engage in community health planning efforts.

### Review of written documents and records

Archival records were reviewed to document the implementation of CCHI in each area. These records included the original grant proposal, county assessments, project and intervention progress reports, county statistical data, and grant proposals that resulted from CCHI resources.

### Participant observation

The seven members of the steering committee were interviewed by the same interviewer to determine how the implementation of the initiative compared to its original intentions. Two of the authors of this report were members of the steering committee and were also directly involved in the entire CCHI process. Our case study narratives draw from their understanding of how implementation of the initiative was influenced by the local resources, needs, priorities, and leadership styles that existed in each county.

### Stakeholder interviews

Semi-structured interviews were conducted with the hospital chief executive officer, the health department director, the CCHI local coordinator, and coalition representatives in each county for a total of 24 interviews. Respondents signed an informed consent form, approved by the health care system's Institutional Review Board. Respondents were asked to describe their role in the project, the role that CCHI had played in the community, the benefits of CCHI, the effectiveness of the intervention model, and the effect of CCHI on local perceptions of the health care system.

### Analysis

Interviews were taped, transcribed, and analyzed using QSR N5 (QSR International Pty Ltd, Melbourne, Australia), a software package that allows for exploration and coding of qualitative data; text search; quantitative assessment of prevalence of key themes; and examination of relationships among key concepts (20). The research team worked collectively through the coding of one interview transcript to identify a framework of themes and concepts. Each researcher then independently reviewed each interview transcript using this framework, and the group agreed upon a common set of themes and categories for coding. The analysis was led by an author not affiliated with the CCHI project; this author coded each transcript and prepared a summary of the key findings. Narrative case reports were prepared for each of the four projects using data from all three sources. The case study materials were again analyzed to draw cross-case conclusions about the characteristics of local communities that define their capacity to engage in community health planning efforts.

## Consequences

### The small rural county

#### Project implementation

CCHI worked with an existing coalition of representatives from the hospital, health department, and local agencies. There was strong collaboration, but membership was limited in scope. Despite recruitment efforts, there were few African American participants and there was limited participation from the local school system and local community health center. Planning was more reactive than proactive because local needs were often overwhelming, but participants maintained a high level of pride, energy, and involvement. One influential leader played a dominant role within the coalition, partly because of repeated turnover in health department leadership during the course of the project. The results of the CCHI community assessment were not highly utilized, and controversy took place over some funding decisions. Much of this stemmed from a perception of CCHI staff as outsiders and a strong concern about preserving local autonomy in decision making. Coalition members felt that the county should determine CCHI funding because it was acutely aware of local needs and had specific programs that needed funding.

#### Local stakeholder reaction

Of the four study sites, this county had the most negative perception of CCHI and maintained a perception of CCHI as "Big Brother." Respondents were less likely to report that the project resulted in improved coalition building, was responsive to local needs, used a flexible approach, or had improved the image of CHS. Respondents indicated that CCHI and county priorities were not in agreement. One stakeholder commented, "I think CCHI needs to rely more on the [coalition]. . . . [T]here was some controversy because decisions were made about grants in Charlotte rather than in [our county]."

However, the majority of respondents indicated that the system of health care was better as a result of CCHI interventions and that the director of CCHI was highly respected. Resources and expertise were the most frequently cited benefits provided by CCHI, with funding for local initiatives clearly identified as the most important aspect. One respondent said, "[The] county is a poor rural county and there are just not that [many] resources here, but CCHI has helped the [group] find resources that [we] did not think [we] could find."

### Midsize rural county

#### Project implementation

CCHI worked with a preexisting local coalition comprised of leaders of the major health and human service organizations who had considerable experience working together to assess community health needs and implement health improvement initiatives. While they were eager to work with CCHI, they clearly wanted their planning and organizational efforts to drive the process and were concerned that CCHI would duplicate local energies. To address this concern, efforts were made to augment and enhance existing assessments with previously unused data and integration of geographic information system mapping technology. Members of the coalition were also concerned about how data would be used, and there was initial difficulty gaining access to state and local health department data. An agreement was reached whereby available funds would primarily support activities specified in the coalition's action plan. This would allow coalition members to expand existing efforts and build a greater community presence.

#### Local stakeholder reaction

Interviews indicated an overall positive perception of the CCHI project. CCHI was described as a facilitator and partner, and respondents in the county were outspoken about CCHI having worked with the county and used a flexible approach. Critical to the success of CCHI was the perception that local autonomy had been preserved, and the community was empowered by CCHI to develop a better health care system**.** One participant commented, "[CCHI has] empowered us not only with resources, but the ability to choose how to use them. . . . CCHI understands our strengths and has allowed us to use those strengths."

The majority of respondents described staff support as a significant benefit of CCHI and reported increased coordination within the county. However, many felt that the needs assessment duplicated existing efforts. Availability of funding for local interventions was the most commonly stated benefit. For example, one respondent offered this statement: "CCHI has helped us go from a virtual organization . . . to a more permanent organization because of our ability to access funding through CCHI and our ability to have a coordinator."

### Urban community

#### Project implementation

This project was unique among the four projects because it built on a community-oriented primary care effort — a coalition of local community residents and health care providers who had begun to define and address community health issues. However, initial distrust of the health care system was a significant challenge for CCHI staff. Residents had questions about the intent of the project and were highly suspicious of CCHI's long-term commitment to continued support of community-based initiatives. Community groups were highly independent and were often distrustful of so-called data-driven issues or needs. There were significant conflicts over how funds would be distributed to the community. However, community organizations were perceived by CCHI as being loosely organized, and there were concerns about their capacity to take on additional responsibilities without significant support. Some of these conflicts improved as the coalition gained experience working collaboratively on the community assessment and interventions.

#### Local stakeholder reaction

Interviews with CCHI stakeholders revealed a surprisingly positive view of CCHI. Issues of trust and independence were a common theme, but it was clear that CCHI had made progress in overcoming the community's original image of the health care system. One stakeholder said, "There was significant community distrust about Carolinas HealthCare Systems' lack of responsiveness to the community needs. I think while some of that is still there, much of it has dissipated based on getting to know one another and discussing some things. I think [CCHI] may have changed the community attitude."

Respondents in this community were the most likely of all sites to feel that CCHI had a direct impact on coalition building and overcoming existing disputes over priorities within the county. The respondents felt that the CCHI model had helped produce community-based care and had provided coalition-building efforts. One commented, "One of the benefits is that CCHI brings another resource to the table, not only in the terms of funding but also in terms of involving community members at the neighborhood level, and getting input, . . . involvement, . . . and creating a sense of ownership for the folks at that level."

### Rural-urban county

#### Project implementation

This county was furthest behind in implementing the CCHI model and struggled to develop a coalition. Some community health leaders questioned the need for a coalition. Among those who saw potential benefit, there was concern about sustainability and commitment. A fiscally conservative political atmosphere had limited collaboration among service organizations to small projects. Because initial attempts to develop an active coalition were not successful, initiatives and projects were developed and produced almost solely by the coordinator. To revitalize the process, a chairperson with local name recognition was identified and a coalition slowly began to form. Based on the findings of the health needs assessment, several community health interventions were developed and funded.

#### Local stakeholder reaction

Similar to the other sites, respondents were positive in their evaluation of CCHI. The majority felt that CCHI had provided needed resources and reported increased collaboration with CCHI. Funding was the most commonly stated benefit. A respondent said, "This past year they allocated about one hundred thousand dollars for grants. . . . There are some new programs and new things that got started because of that funding."

Most respondents felt that CCHI had played a major role in developing coalitions, although some were critical of the coordinator for not facilitating further collaboration. Uncertainty and understanding of the coordinator's role was identified as a particular challenge to the process. Several respondents reported that the coordinator role faced positional challenges: "She has been strained in many ways. It's been a one-man operation. . . . She's been by herself . . . and had very little assistance . . . and hasn't had a lot of resources."

## Interpretation

Our interviews and case studies provide useful information on how to engage communities and large health care systems in ways that go beyond the medical care model. Within the CCHI communities, historical experiences with the health care system were highly significant. Communities were suspicious of CCHI's intentions and concerned that the health care system would attempt to set agendas with little community input. Communities were reluctant to share certain health care data, and they were concerned that existing efforts would be duplicated.

These concerns were settled to varying degrees as working relationships evolved and resources and services became available that were seen to supplement and support local efforts. Local coordinators were important in increasing receptivity. They played a key role in helping to establish all the projects because of their personal and professional connections and knowledge of local resources and efforts, and they were perceived as valuable resources in all the sites. Attempts to institutionalize their role occurred in two counties, and a part-time position was created in a third.

The communities involved in the CCHI project represented diverse economic backgrounds. Two communities had experienced significant population migration and loss of local industry. One had high levels of poverty and unemployment. These communities had strong senses of need and discouragement over seemingly intractable socioeconomic situations. CCHI funding for local demonstration projects was clearly seen as the most significant benefit of the project. In the transitional rural community, proximity to a prosperous urban center had stimulated the local economy. However, a conservative political climate had limited the community's momentum to expand local efforts or pursue progressive programs. In this community, much of CCHI's efforts were geared toward building capacity by offering structure and support.

Overall, the project was effective in advancing local efforts in all four settings by increasing local capacity and by supporting comprehensive approaches to chronic disease prevention. Table 2 summarizes the main outcomes of the project. Specific changes and accomplishments are documented for three core areas: increases in breadth and diversity of participation in public health planning, support of local structure, and identification of additional funding sources for sustainability. While many of these changes in local capacity may have occurred independently of this project, it is clear from the comments of local stakeholders that the resources provided by CCHI contributed to and often served as a catalyst for needed action.

Funding was provided to each county for a wide range of interventions related to health promotion and chronic disease prevention. Evaluation of the interventions has been qualitative and process oriented as measurable improvements in behavior or physiologic indicators are unlikely to be seen so soon. The goal of the local funding component was to promote and support a full spectrum of health promotion strategies using a multilevel approach. Table 2 documents interventions related to organizational development, individual behavior change, and changes in policy and environment in each county. This approach was intended to provide a broad foundation of health promotion infrastructure that could eventually lead to sustainable improvements in chronic disease indicators.

In four case studies, we have presented the steps, challenges, and opportunities faced by a large health care system seeking to create collaborative health planning projects to impact chronic disease at the community level. Our approach included six components that appeared to be relatively well accepted in all four sites. Distrust and the need for local autonomy were important areas of conflict. The project was successful in increasing local capacity and supporting multilevel interventions to prevent chronic disease. Our experience should be particularly instructive to large health care systems in developing and supporting local community health initiatives.

## Figures and Tables

**Table 1:** Characteristics of Four Communities Studied as Part of Regional Health Care System Partnership, North Carolina, 2001

**Characteristic**	**Small Rural County**	**Rural-urban County**	**Midsize Rural County**	**Urban Community**	**North Carolina**
**Setting**	Very rural — small towns	Transitional/ growing — midsize towns	Rural — midsized towns	Urban — underserved neighborhoods	Not applicable
**Coalition status at beginning of project**	Existing	None	Existing	None	Not applicable
**Population^a^**	25,275	123,677	96,287	18,614	8,049,313
**Percent 65 years and older**	14.4	9.0	13.5	13.2	12.0
**Percent white**	49.5	82.8	76.8	5.6	72.1
**Percent African American**	48.6	12.5	20.9	88.0	21.6
**Percent Hispanic/Latino**	0.8	6.2	1.5	4.2	4.7
**Percent below poverty level**	17.8	8.1	13.3	25.0	12.3

**Chronic Disease Indicators**					

**Age-adjusted mortality rates^b^**					

**Heart Disease**	312	280	291	262^c^	259
**Stroke**	72	67	77	99^c^	75
**Cancer**	198	192	208	183^c^	204

**Hospital discharge rate** d					

**Asthma**	82	56	62	90	55
**Hypertension**	465	296	415	440	362
**Diabetes**	232	152	209	214	182

a Source: Statistical Abstract of the United States 2000 (15).

b Per 100,000 population. Source: Certificate of Death Data files, 2001 (16).

c Source: Certificate of Death Data files, 1995 (17).

d Per 100,000 hosptial discharges. Source: Hospital Discharge Data, North Carolina, 2001 (18).

**Table 2:** Intervention Matrix for Four Communities Participating in Regional Health Care System Partnership, North Carolina, 2001

	**Capacity Building**		

	**Participation**	**Structure**	**Sustainability**
**Small rural county**	Funding for school teacher health promotion training.	Integrated nontraditional data sources for community health assessment process.	Leveraged private foundation funding for community health projects.
**Midsize rural county**	No events documented.	Enhanced community assessment process that integrates multiple agency assessments into one process.	Leveraged private foundation and state funding for community health projects.
**Rural-urban county**	Latino outreach initiative to address health disparities.	Established community health coalition. Integrated nontraditional data sources for community health assessment.	No events documented.
**Urban community**	Developed community/ primary care site partnership. Developed YMCA partnership.	Established cardiovascular disease/diabetes coalition. Partner community group received 501(c)3 designation.	Leveraged national CDC health disparities grant. Leveraged county funding for local neighborhood farmers’ market.

	**Funded Chronic Disease-Related Interventions**		

	**Organizational Development**	**Individual Behavior Change**	**Environmental/Policy Change**
**Small rural county**	Health Education Improvement program targeting teachers of grades 6–12.	Mobile wellness and screening program in outlying areas. Comprehensive parent education program on adolescent health.	Parish nurse program.
**Midsize rural county**	Alzheimer's support and referral project.	No events documented.	Faith-based projects including parish nurse program and congregational heart-disease health promotion teams.
**Rural-urban county**	Emergency Medical Services “Living Safely” program for cardiovascular disease and stroke early intervention.	Community nutrition and exercise program in local faith communities.	Latino Outreach and Health Access Initiative to address health disparities. Automatic defibrillator community awareness project.
**Urban community**	Support to develop an action plan for a county health disparities task force. Staff support for *REACH 2010* project.	Chronic disease education and screening programs in community sites. Sponsorship of adolescent health promotion and wellness camp.	Staff support for farmers market.
